# KIAA1199 is a secreted molecule that enhances osteoblastic stem cell migration and recruitment

**DOI:** 10.1038/s41419-018-1202-9

**Published:** 2019-02-12

**Authors:** Li Chen, Kaikai Shi, Thomas Levin Andersen, Weimin Qiu, Moustapha Kassem

**Affiliations:** 1 Department of Endocrinology and Metabolism, Endocrine Research Laboratory (KMEB), Odense University Hospital and University of Southern Denmark, 5000 Odense, Denmark; 2 Department of Clinical Cell Biology, Vejle Hospital-Lillebaelt Hospital, Institute of Regional Health Research, University of Southern Denmark, 7100 Vejle, Denmark; 3 The Danish Stem Cell Center (DanStem), University of Copenhagen, 2200 Copenhagen, Denmark; 4 Stem Cell Unit, Department of Anatomy, Faculty of Medicine, King Saud University, Riyadh, Saudi Arabia

## Abstract

Factors mediating mobilization of osteoblastic stem and progenitor cells from their bone marrow niche to be recruited to bone formation sites during bone remodeling are poorly known. We have studied secreted factors present in the bone marrow microenvironment and identified KIAA1199 (also known as CEMIP, cell migration inducing hyaluronan binding protein) in human bone biopsies as highly expressed in osteoprogenitor reversal cells (Rv.C) recruited to the eroded surfaces (ES), which are the future bone formation sites. In vitro, KIAA1199 did not affect the proliferation of human osteoblastic stem cells (also known as human bone marrow skeletal or stromal stem cells, hMSCs); but it enhanced cell migration as determined by scratch assay and trans-well migration assay. KIAA1199 deficient hMSCs (KIAA1199^down^) exhibited significant changes in cell size, cell length, ratio of cell width to length and cell roundness, together with reduction of polymerization actin (F-actin) and changes in phos-CFL1 (cofflin1), phos-LIMK1 (LIM domain kinase 1) and DSTN (destrin), key factors regulating actin cytoskeletal dynamics and cell motility. Moreover, KIAA1199^down^ hMSC exhibited impaired Wnt signaling in TCF-reporter assay and decreased expression of Wnt target genes and these effects were rescued by KIAA1199 treatment. Finally, KIAA1199 regulated the activation of P38 kinase and its associated changes in Wnt-signaling. Thus, KIAA1199 is a mobilizing factor that interacts with P38 and Wnt signaling, and induces changes in actin cytoskeleton, as a mechanism mediating recruitment of hMSC to bone formation sites.

## Introduction

Human osteoprogenitor cells, also known as human skeletal stem cells, marrow stromal or mesenchymal stem cells (hMSCs), represent a population of non-hematopoietic cells that exist at different locations within the bone marrow near eroded surfaces and can differentiate into mature osteoblastic bone forming cells^1,2^. The initiation of in vivo bone formation during skeletal remodeling and bone regeneration during fracture healing depend on the mobilization of sufficient number of osteoprogenitor cells to future bone formation sites^1^. This critical recruitment is impaired during aging and in metabolic bone diseases, including osteoporosis^1,3^.

As bone remodeling takes place asynchronously in the skeleton, the coupling of bone formation to resorption is tightly orchestrated by local coupling factors. These coupling factors are believed to mobilize osteoprogenitor cells from their niche, and recruit them to eroded surface prior to initiation of bone formation^1^. However, the identity of these factors is under investigation and currently only few have been identified and shown to be produced by osteoclastic, osteoblastic cells or other cells in the hematopoietic microenvironment^4^.

From a translational perspective, hMSCs have been employed in an increasing number of clinical trials for enhancing bone formation and tissue regeneration^2^. However, systemically infused hMSCs exhibit poor homing to the injured tissues^5,6^ and the majority of the cells are trapped in the lungs with very few cells reaching and engrafting in the skeleton^7,8^. To achieve clinical goals of using hMSCs in therapy, there is a need for identifying molecules and factors that enhance hMSCs migration and motility^9–11^.

Several factors have been identified to ‘mobilize’ hematopoietic stem cells out of their niche as the first step for induction of differentiation^12^, but very few factors have been reported to enhance hMSCs mobilization from their bone marrow niche. Substance P has been reported to mobilize a subgroup of bone marrow stromal cells with MSC-like phenotype^13^. Also, following bone fracture, the number of circulating human MSC-like cells increased^14^ suggesting that changes in bone microenvironment following bone fracture, release osteoprogenitor cells mobilizing factors that are yet to be identified.

We have previously performed a global quantitative proteomic studies on hMSCs secretome, and identified a number of secreted factors which regulate MSCs lineage allocation, differentiation and functions^15^, e.g., Legumain (LGMN) and Collapsin Response Mediator Protein 4 (CRMP4)^16,17^. Among the identified factors, KIAA1199 was found to be highly expressed by hMSCs in vitro and in vivo but its function in hMSCs biology is not known.

KIAA1199, also named as CEMIP (cell migration inducing protein), is expressed from a gene located on chromosome 15q25.1 and encodes 150 kDa protein^18^ with N-terminal secretion signal peptide. KIAA119 has a PbH1 domain consisting of parallel beta-helix repeats, which is predicted to function in polysaccharide hydrolysis^19^, G8 domain containing eight conserved glycine residues and five repeated beta-strand pairs and one alpha-helix^20^, and two GG domains consisting of seven beta-strands and two alpha-helices^21^. Many G8-containing proteins are integral membrane proteins with signal peptides and/or transmembrane segments, suggesting that KIAA1199 is a secreted factor that plays a role in extracellular ligand binding and processing. The biological role of KIAA1199 has been studied in cancer biology and a number studies has demonstrated high expression levels in cancer cell lines and its association with invasive and metastatic disease^22,23^.

In the present study, we examined regulatory role of KIAA1199 in hMSCs migration, as well as its cellular and molecular mechanism of action. We observed that KIAA1199 is expressed in osteoprogenitor cells and enhances their migration abilities through regulation of cell shape, actin cytoskeletal dynamics and regulation actin depolymerizing factors Cofilin1 (CFL1), LIM domain kinase 1 (LIMK1) and Destrin (DSTN). Furthermore, KIAA1199 promotes cell migration by cooperative activation of canonical Wnt and p38/MAPK signaling.

## Material and methods

### In situ hybridization

Formalin-fixed, decalcified and paraffin-embedded bone specimens from eight human controls were included in situ hybridization analysis. Four of the human specimens were diagnostic iliac crest biopsies obtained from control patients formerly under investigation for a hematological disorder, as previously described^24^, while the remaining four human specimens were from the proximal femur of coxa valga patients, as previously described^1^. The study is approval by the Danish National Committee on Biomedical Research Ethics (S-20070121 and S-20120193). Paraffin sections (3.5-µm thick) were in situ hybridization using an enhanced version of the RNAScope 2.5 high definition procedure (310035, ACD Bioscience, Attikis, Greece). Sections were rehydrated, deparaffinized and pretreated as previously^24^, and hybridized overnight at 40^°^C with 20-ZZ-pair probes binding either the X-Y region of the human KIAA1199 mRNA diluted 1:1 in probe diluent (Cat No. 449819, ACD Bioscience). A negative control only hybridized with probe diluent was included. The amplification was conducted according to the instructions provided by the manufacturer. The horse radish peroxidase was further enhanced with digoxigenin (DIG)-labeled tyramide (NEL748001KT, PerkinElmer, Skovlunde, Denmark), which was labeled with alkaline-phosphatase-conjugated sheep anti-DIG FAB-fragments (11093274910, Roche, Hvidovre, Denmark) and visualized with Liquid Permanent Red (DAKO, Glostup, Denmark). Finally, the sections were counterstained with Mayer’s Haematoxylin and mounted with Aqua-Mount.

### Cell culture

As a model for human osteoprogenitor cells, we employed the telomerized hMSCs (hMSC-TERT) line that was established in our laboratory by stable overexpression of human telomerase reverse transcriptase gene (hTERT)^25^. hMSC-TERT expresses all known markers of osteoprogenitor cells including the ability to form bone when implanted in vivo^25^. To simplify, hMSC-TERT will be referred to as hMSCs. Cells were cultured in Minimum Essential Medium (MEM) with 10% fetal bovine serum (FBS) and penicillin-streptomycin (P/S) (1%). All reagents were purchased from Life Technologies (Nærum, Denmark). Cells were incubated in 5% CO_2_ incubators, 37 °C and humidity of 95%.

### Cell transfection and infection

hMSCs and HEK293T cells were cultured until 70–80% confluent and transfected by small interfering RNA (siRNA) or constructed plasmids using Lipofectamine 2000 (Thermo-Fisher Scientific, Roskilde, Denmark) according to the manufacturer’s instructions. siRNAs for specific genes or control non-targeting siRNA were all purchased from Silencer^®^ Selector siRNA library from Thermo-Fisher Scientific. The KIAA1199 (Gene ID: NM_001293298.1) expressing plasmid construct was produced with pLVX-mCMV-ZsGreen-Puro vector (BioWit, Shenzhen, China). Plasmid DNA preparations were made from over-night cultures of individual colonies using the Plasmid MidiPrep System (Qiagen, Stockach, Germany) following the manufacturer’s protocol.

Lentiviruses were made by transfecting packaging cells (HEK293T) with a three-plasmid system: DNA for transfections was prepared by mixing plasmids for KIAA1199, pVSVg, and psPAX2 with Lipofectamine® 2000 Reagent according to the manufacturer’s instructions (Thermo-Fisher Scientific, Roskilde, Denmark). Lentiviral supernatants were collected at 36 h post-transfection and added polybrene to 8 μg/ml, then immediately used to infect target cells. Cells were incubated for 24 h and the media was changed to remove virus particles. Transduced cells were cultured in the presence of 1.0 μg/ml puromycin to select for positive cells for one month. A control cell line was generated by transducing hBMSC-TERT with virus containing the empty pBABE-puro vector.

### Quantitative real-time PCR (qRT-PCR)

RNA from cells was isolated by TRIzol® according to the manufacturer’s instructions (Thermo-Fisher Scientific, Roskilde, Denmark). The first strand complementary DNA was synthesized from 1 µg total RNA by Revert aid cDNA kit (Sigma, Copenhagen, Denmark). RT-qPCR was performed by ABI StepOne^TM^ Real-TIME PCR machine with SYBR green (Applied Biosystems, Roskilde, Denmark). The primers of genes used in the study were listed in supplemental Table 1. The data was normalized to geometric means of the several reference genes and analyzed by a comparative CT method where ΔC_T_ is the difference between the CT values of the target and the geometric mean of the reference genes.

**Table 1:** Primers for qRT-PCR analysis

Gene ID	Gene name	Gene sequence ID	5’ → 3’ Forward primer	5’ → 3’ Reverse primer	length (bp)
KIAA1199	Cell migration-inducing and hyaluronan-binding protein	NM_001293298.1	TCTTTGGGCCACTGCTTCTTCACG	GTCTTGCCTGGGCTTGGGGATGTA	153
NKD1	Naked cuticle Homolog 1	NM_033119.4	GATGGAGAGAGTGAGCGAACC	CATAGATGGTGTGCAGCAAGC	161
DKK2	Dickkopf WNT signaling pathway inhibitor 2	NM_014421.2	AGCATCTTAACCCCTCACATCC	TTTCCAGCCCATGAGAACC	264
TNFRSF19-2	Tumor necrosis factor receptor superfamily member 19	NM_148957.3	CATTTCATCTCCCTGCTCG	GCCACATTCCTTAGACAACTCC	357
Axin2	Axin-like protein 2	NM_004655.3	TACACTCCTTATTGGGCGATCA	TTGGCTACTCGTAAAGTTTTGGT	128
ATCB	Beta-actin	NM_001614.3	ATTGGCAATGAGCGGTTCCG	AGGGCAGTGATCTCCTTCTG	192
B2M	Beta 2-microglobulin	NM_004048.2	CCTTGAGGCTATCCAGCGT	CCTGCTCAGATACATCAAACATG	510
GAPDH	Glyceraldehyde-3-phosphate dehydrogenase	NM_002046	GGCGATGCTGGCGCTGAGTAC	TGGTTCACACCCATGACGA	130

### Collection of conditioned medium

Cells were cultured as required at the specified time point, and washed with PBS and incubated in serum-free medium for 8–12 h. The conditioned medium (CM) was then collected. Floating cells were spun down at 250×*g* for 5 min. The supernatant was kept in –80 °C. For Western blot analysis at protein in CM, CM was concentrated (1:20) by centrifugation at 3000×*g* for 30–45 min with Amicon Ultra Centricons (10 kDa NMWL) (Millipore, Hellerup, Denmark).

### Western blot analysis

Western blot analysis was performed as described previously^26^. The cells were washed in cold PBS twice and lysed in RIPA buffer (Thermo-Fisher Scientific, Roskilde, Denmark) supplemented with protease inhibitors (Roche, Hvidovre, Denmark) for 30 min in cold room. Samples were centrifuged at 12,000 r.p.m., 4 °C for 10 min. Protein concentrations were determined with a BCA kit (Thermo-Fisher Scientific, Roskilde, Denmark), and equal amounts (30 µg) of proteins were loaded on a polyacrylamide gel (Thermo-Fisher Scientific, Roskilde, Denmark). Blotted nitrocellulose membranes were incubated overnight with primary antibody at 4 °C, and were developed after 1 h incubation with secondary anti-rabbit horseradish peroxidase-conjugated antibody (Santa Cruz Biotechnology, Heidelberg, Germany) using an ECL Western blotting kit (GE Healthcare, Brøndby, Denmark) and Kodak films. Antibodies for KIAA1199 was purchased from ProteinTech, Manchester, UK; Antibodies (total or phosphor) specific for CFL1, LIMK1, p38, GSK3β and beta-catanin were obtained from Cell Signaling (Herlev, Denmark); antibodies for DSTN and actin were bought from Sigma-Aldrich (Copenhagen, Denmark). All antibodies were used at 1:1000 dilutions except actin antibodies (1:2500).

### Cell counting and cell viability

The cells were washed by PBS, trypsinized, and stained with 0.4% Trypan Blue (1:1 V/V) and counted using a hemocytometer. Cell viability was determined by Cell Titer-Blue^®^ cell viability assay according to the manufacturer´s instructions (Promega Biotech AB, NACKA, Sweden). In briefly, cells were cultured in 96-well plates and 20 µl/well of CellTiter-Blue Reagent was added and incubated for 1 h at 37 °C. Fluorescence at 560/590 nm by FLUO Star Omega Plate Reader (BMG Laboratories, Ortenberg, Germany) was determined.

### TCF luciferase reporter assay

TCF-dependent transcriptional activity was studied in hMSC-TERT transduced with TCF promoter constructs along with Renilla luciferase reporter gene (50:1 ratio) as described previously^27^. After 48 h of siRNA transfection, firefly and Renilla luciferase activities were measured using the Dual-Glo Luciferase Assay System (Promega Biotech AB).

### In vitro scratch assay

The confluent monolayer of cells was scratched with a 1000-μl pipette tip smoothly to induce a gap without cell detachment, and the cells were cultured for 8–48 h. Images were collected at the same positions of the plate at different time points post-scratching. Images were quantified using Image-J software that measured the migrated area.

### Trans-well migration assay

Cells were cultured to 70–80% confluence and starved for 18–20 h in serum-free LG DMEM (Life Technologies, Tastrup, Denmark). Transwell migration assay was performed using 12 mm Millicell^®^ inserts (Millipore). Briefly, adding 5% FBS MEM or specific conditional medium in the lower chamber of well, test cells were plated in the upper chamber in 0.2% FBS DMEM medium and incubated at 37 °C for 16 h. The cells on the surface of the upper chamber were removed by cotton swabs; the membranes in the inserts were removed and fixed by formalin buffer (Sigma-Aldrich) for 5 min, stained in Hemacolor staining solution (Merck, Germany) for 15 min. Transfer the membranes to glass slide (Menzelgläzer, Germany) and scan the whole membrane by a Lecica DM4500 microscope (Olympus, UK). The numbers of migrated cells or sizes of membrane area covered by the cells were determined by Surveyor Turboscan Mosaic acquisition imaging system (Objective Imaging, Cambridge, UK).

### Single cell morphology analysis by Operetta high content imaging system

Single cell morphology analysis was performed using Operetta CLS^TM^ High Content Imaging system (PerkinElmer, Skovlunde, Denmark) as we described in a previous study^28^. Briefly, the cells were trypsinized and seeded as 1000 cell/well into clear bottom 96-well CellCarrier™ microtiter plates (PerkinElmer, Denmark). The cells were fixed in 4% paraformaldehyde for 10 min, washed with PBS, stained with DAPI (Sigma-Aldrich, D8417) and Phalloidin-TRITC (Sigma-Aldrich®, P1951). Fluorescent images were analyzed at × 10 and × 40 magnification using Harmony High Content Imaging and Analysis Software (PerkinElmer, Denmark). All pictures were acquired with the same contrast and brightness parameters, and data analysis performed by same parameters setting.

### Statistical analysis

Data are expressed as the mean and standard deviation (SD). Student’s t-test was used to assess differences between two groups, and one-way analysis of variance (ANOVA) was used for statistical testing involving more than two groups. *P* < 0.05 considered to be significant. *n* = number of independent experiment and each experiment was repeated as least 3 times.

## Results

### KIAA1199 is expressed in osteoprogenitor cells in human bone biopsies

**Fig. 1:**
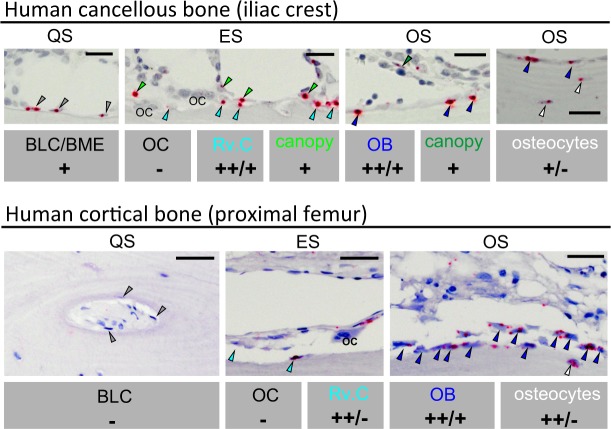
KIAA1199 is expressed in osteoprogenitor cells colonizing future bone formation sites in human bone. Four human iliac crest bone specimens were in situ-hybridized for KIAA1199. KIAA199 expresses in reversal cells (Rv.C–light blue arrowheads), i.e., progenitor cells, colonizing the eroded surfaces (ES-future bone formation sites), the bone lining cells (BLC), bone marrow envelope (BME) (gray arrowheads) above quiescent surfaces (QS), as well as canopy cells (green arrowheads) above eroded surfaces (ES) that reported to be a local source of osteoprogenitor cells. KIAA1199 expression was also clear present in mature osteoblasts (OB–dark blue arrowheads) on osteoid surfaces (OS) and in some osteocytes, and absent in osteoclasts (OC). The overall expression level in each cell type is indicated below their illustrations: - no expression; + low expression, + + medium expression. Scale bar: 40 µm

Our previous study had identified KIAA1199 as a secreted factor by human osteoblastic stem cells, and its secretion is enhanced during osteoblast differentiation in vitro^15^. To corroborate the cellular expression of KIAA1199 in the bone marrow microenvironment in vivo, we performed in situ hybridization analysis of adult human iliac crest bone biopsies that revealed KIAA1199 expression was localized to bone formation sites during bone remodeling and expressed specifically in immature and mature osteoblastic cells along bone surfaces in both cancellous and cortical bone (Fig. 1). KIAA1199 expression was abundant in the osteoblastic reversal cells (Rv.C) recruited to the eroded surfaces (ES), which are the future bone formation sites (Fig. 1). These cells have recently been reported to reflect motile osteoprogenitor cells which differentiate into bone-forming osteoblastic cells when reaching a critical cell density^29^. Moreover, KIAA1199 expression was present in osteoblastic bone lining cells (BLCs) and bone marrow envelope (BME) above quiescent surfaces, as well as osteoblastic canopy cells above eroded and osteoid surfaces (Fig. 1). These cells have been reported to be local source of osteoprogenitor cells (hMSCs) which are recruited to the eroded and osteoid surfaces during bone remodeling^29^. KIAA1199 expression was rich present in mature bone-forming osteoblast, but exhibited limited expressed in osteocytes and was absent in osteoclasts (Fig. 1).

### KIAA1199 regulates hMSCs cell migration

**Fig. 2:**
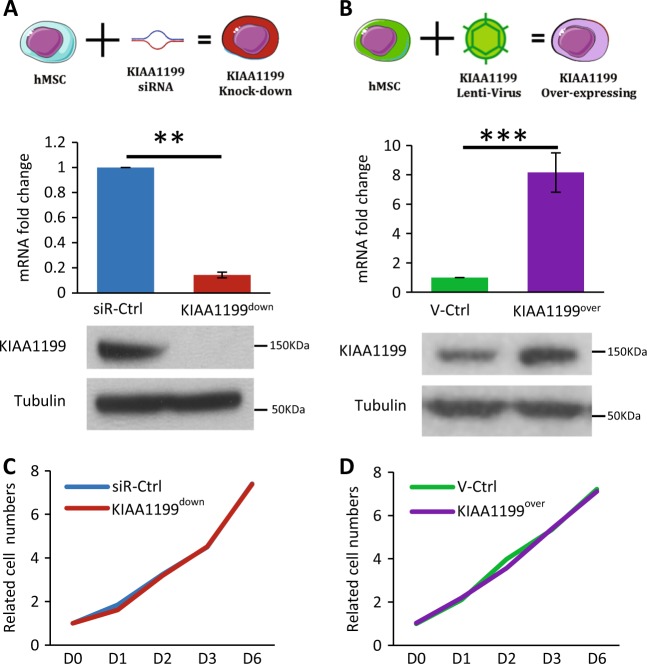
Establishing of KIAA1199 deficient or overexpressing human bone marrow skeletal (stromal) stem cells (hMSCs). **a** KIAA1199 deficient hMSCs (KIAA1199^down^) were obtained by transfecting the cells with a specific siRNA for KIAA1199 (siR-KIAA1199) or with non-target siRNA control (siR-Ctrl); (**b**) KIAA1199-overexpression hMSCs (KIAA1199^over^) were established using a lentiviral transduction and, corresponding control was established by parallel infected by lentiviral vehicle (V-Ctrl). Expression levels of KIAA1199 in cells were determined using qRT-PCR and Western blot analysis (**a, b**). **c–d** Cell proliferation was determined by cell counting and cell viability assay

**Fig. 3:**
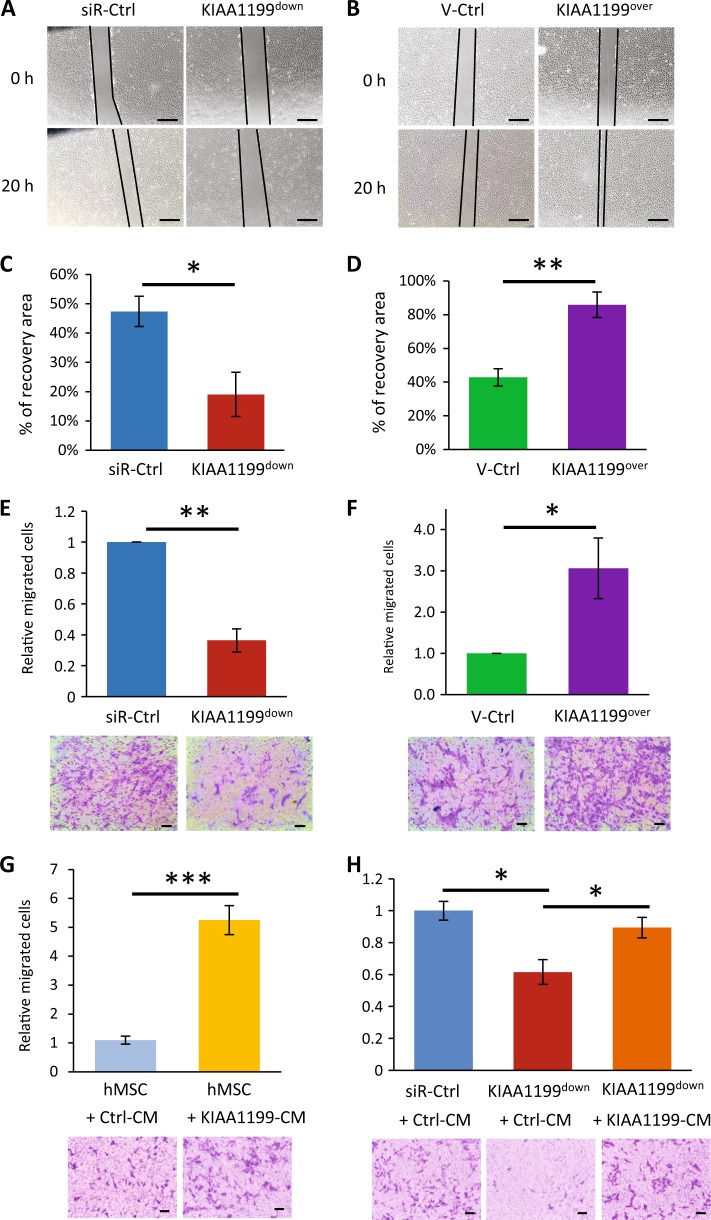
KIAA1199 regulates human bone marrow skeletal (stromal) stem cells (hMSCs) cell motility and migration. **a–b** In vitro scratch assay was performed in hMSC-KIAA1199^down^ or hMSC-KIAA1199^over^ cells. Photomicrographs were obtained at the same position of culture dish after 20 h incubation in 0.2% fetal bovine serum (FBS) cultured medium, Scale bar: 500 µm. **c–d** The ability of the cells to ‘heal the wound’ (migrated area) was determined as a ratio of cell covered area per total area by Image-J^®^ program (*n* ≥ 3). **e–f** Boyden Chamber trans-well cell migration assay were performed in hMSC-KIAA1199^down^ or hMSC-KIAA1199^over^ and cell migration was measured after 16 h. The migrated cells were calculated by Image-J program (*n* ≥ 3). **g** hMSCs were placed in the upper chamber of Boyden chamber system and Lower chambers were filled with conditional mediums (CM) from KIAA1199 overexpression cells (KIAA1199-CM) or its corresponding vehicle control cells (Ctrl-CM). Cell migration was measured after 16 h. **h** KIAA1199 deficient hMSCs (KIAA1199^down^) and control cells (siR-Ctrl) were placed in upper chamber of Boyden chamber system, Ctrl-CM or KIAA1199-CM were added into lower chamber. Cell migration was measured after 16 h. The migrated cells were calculated by Image-J program (*n* ≥ 3). Data represent mean ± SD. *P < 0.05, **P < 0.01, ***P < 0.001. Scale bar for pictures: 500 µm

Based on the in vivo localization of KIAA1199, we studied its role in hMSCs migration and motility. hMSCs represent a population of stem and osteoprogenitor cells that express osteoblastic markers and can form mineralized matrix in vitro and form bone when implanted in vivo^25^. We established two hMSCs cell lines: KIAA1199 deficient (KIAA1199^down^) and KIAA1199 overexpressing (KIAA1199^over^) hMSCs. Inhibition of KIAA1199 expression was performed by specific siRNA and resulted in 90% reduction of expression evidenced by qRT-PCR and Western blot analysis (Fig. 2a), and the reduced levels of KIAA1199 lasted for more than 12 days (data not shown). KIAA1199^over^ cells were created by lentivirus-mediated gene transfer that resulted in 8 fold higher stable expression of KIAA1199 compared to vector infected control (Fig. 2b). Changes of KIAA1199 expression in hMSCs did not affect cell proliferation (Fig. 2c&d). To study cell migration and motility, we employed two independent assays: in vitro scratch assay that examines cell migration in presence of intact cell–cell interactions and thus mimics migration of cells in vivo^30^ and Boyden chamber transwell migration assay testing the cell migration in suspension^31^. In scratch assay, KIAA1199^down^ exhibited reduced cell motility, as well as reduction of the area covered by the cells, compared with non-target siRNA control cells (siR-Ctrl) (Fig. 3a&c). On the other hand, KIAA1199^over^ showed enhanced cell motility and complete cell coverage of the scratch area when compared to vehicle control hMSCs (V-Ctrl) (Fig. 3b&d). In Boyden Chamber Trans-well migration assay, KIAA1199^down^ exhibited a significant decrease in transwell cell migration (Fig. 3e) and the opposite effect was detected for KIAA1199^over^ (Fig. 3f). Similar result also got in human primary culturing human bone marrow cells: knocking down of KIAA1199 by its specific siRNA inhibited cell migration as the hMSC-KIAA1199^down^ cell line (Fig. S1). Since KIAA1199 is detected as a secreted factor in colorectal epithelial cells^32^ and it has also been reported to be present within the endoplasmic reticulum (ER)^33^. We detected KIAA1199 is expressed and secreted in hMSCs (Fig. S2), and we further studied whether the secreted form of KIAA1199 enhances cell migration. Adding conditioned medium from KIAA1199^over^ (KIAA1199-CM) enhanced cell migration compared to control conditional medium (Ctrl-CM) (Fig. 3g) and also rescued the reduced migration in KIAA1199^down^ hMSCs (Fig. 3h).

### KIAA1199 regulates hMSCs cell morphology and actin cytoskeleton organization

**Fig. 4:**
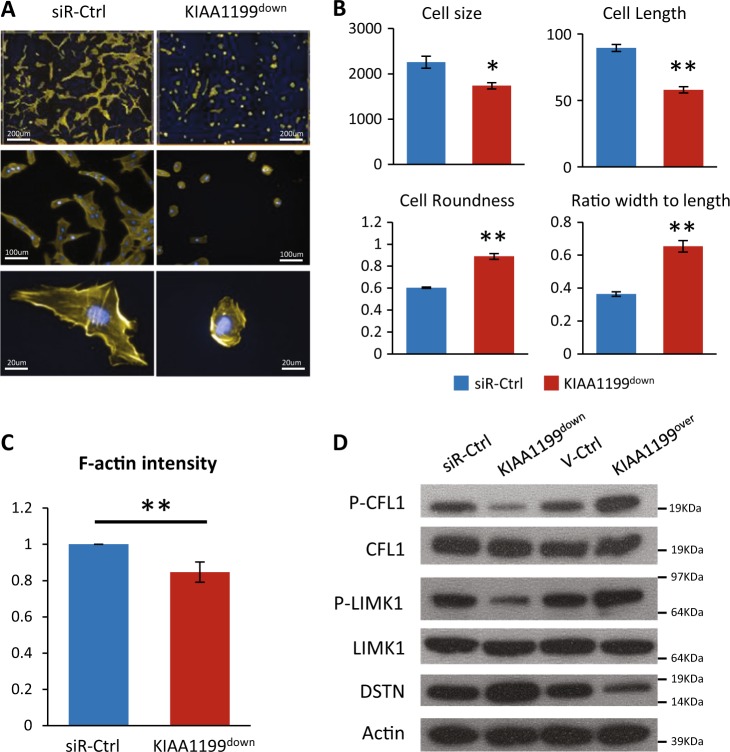
KIAA1199 regulates cell morphology and actin cytoskeleton organization. Human bone marrow skeletal (stromal) stem cells (hMSCs) were cultured and transfected with siRNA specific for KIAA1199 or non-target control siRNAs to obtain KIAA1199 deficient hMSCs (KIAA1199^down^) and the corresponding control cells (siR-Ctrl). **a** The cells were transferred and seeded for 16 h, stained with TRITC-phalloidin and DAPI. Images were acquired on the Operetta® high content image system using ×10 or ×40 objective. **b** Parameters of cell morphology, including cell size (area) (µm^2^), cell length (µm), cell width (µm), cell roundness, and cell ratio (width to length) were analyzed by the Harmony^®^ software. **c** The staining intensity of F-actin was analyzed by the Harmony^®^ software for phalloidin stained F-actin. *N* ≥ 3, *P < 0.05, **P < 0.01. **d** Actin depolymerizing factors involved in actin cytoskeletal dynamics: CFL1, LIMK1 and DSTN, were measured by Western-blot analysis in KIAA1199^down^ or KIAA1199^over^ hMSCs

Cell motility is associated with changes in cell morphology^34^. Thus, we examined the effect of KIAA1199 deficiency on hMSCs morphology employing high content imaging by the Operetta^TM^ high-content image system. As shown in Fig. 4a, hMSCs-KIAA1199^down^ exhibited changes in cell morphology from fibroblast-like to rounded morphology with reduced cell size and cell length, increased cell roundness, altered the ratio of cell width to length (Fig. 4b). Previous studies have shown that cytoskeletal changes in terms of polymerization filamentous actin (F-actin) and depolymerization to globular actin (G-actin) mediates morphological changes affecting cell motility and migration^35,36^. We observed that F-actin staining intensity was significantly reduced in KIAA1199^down^ (Fig. 4c) and this was associated with increased levels of DSTN, decreased the phosphorylation of CFL1 and LIM domain kinase 1 (LIMK1) that are factors controlling actin polymerization and depolymerization (Fig. 4d). The opposite changes were observed in KIAA1199^over^ that exhibited down-regulation of DSTN and up-regulation of P-CFL1 and P-LIMK1 (Fig. 4d).

### Wnt and p38 signaling mediate KIAA1199-enhanced cell motility

**Fig. 5:**
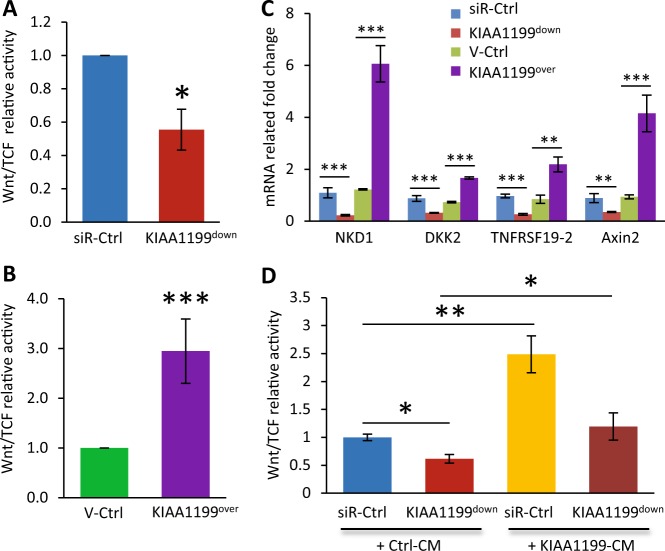
KIAA1199 regulates canonical Wnt signaling in human skeletal (stromal) stem cells (hMSCs). **a–b** Wnt/TCF-luciferase-reporter was transduced into hMSCs deficient in KIAA1199 (KIAA1199^down^) that were created by siRNA-mediated knock-down and control cells transfected with non-target siRNA (siR-Ctrl) (**a**); or transduced into hMSCs overexpressing KIAA1199 (KIAA1199^over^) that was created by lentiviral transduction and corresponding control (V-Ctrl) was established by parallel infected by lentiviral vehicle (**b**). In presence of 50% Wnt3a condition medium induction, TCF-Luciferase activity was measured after 24 h incubation, firefly luciferase was normalized to parallel renilla luciferase activities in the same sample using the Dual-Glo Luciferase Assay System. **c** The mRNA expression levels of canonical Wnt signaling target genes NKD1, DKK2, TNFRSF19-2, and Axin2 were determined by qRT-PCR and normalized to internal controls. **d** KIAA1199^down^ and control cells were cultured in control conditional medium (Ctrl-CM) or KIAA1199 conditioned medium (KIAA1199-CM), with Wnt3a stimulation, TCF-Luciferase activity was measured after 24 h incubation, firefly luciferase was normalized to parallel renilla luciferase activities in the same sample using the Dual-Glo Luciferase Assay System. At least three independent experiments were preformed, data presented as mean ± SD, *P < 0.05, **P < 0.01, ***P < 0.001

**Fig. 6:**
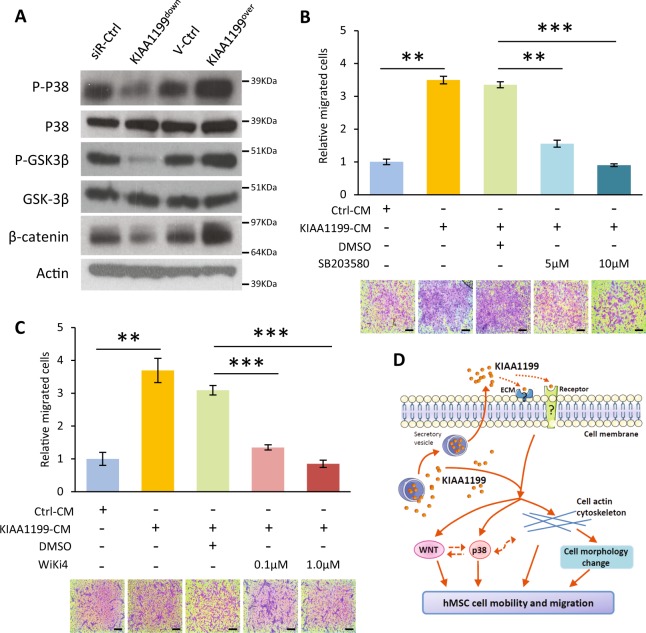
Wnt and p38 signaling are involved in the regulation of KIAA1199 at cell migration in hMSCs. **a** KIAA1199^down^ or KIAA1199^over^ were starved for 6 h, and stimulated with 5% FBS medium for 15 min. Signaling pathways were analyzed by Western-blot analysis. **b** hMSCs were pretreated with P38 inhibitor (SB203580, 5 µM or 10 µM) for 2 h and trans-well migration assay was performed in the absence or presence of KIAA1199 conditioned medium (CM). **c** hMSCs were pretreated with Wnt signaling inhibitor (Wiki4, 0.1 µM or 1 µM) for 2 h and trans-well migration assay was performed in the absence or presence of KIAA1199 conditioned medium (CM). At least three independent experiments were preformed, data presented as mean ± SD, **P < 0.01, ***P < 0.001. Scale bar for pictures: 200 µm. **d** Mechanisms model for the regulation of KIAA1199 in hMSCs mobility and migration

Previous studies suggested that Wnt signaling is important in regulation of stem cell migration^37,38^. To examine the interaction of Wnt/β-catenin signaling with KIAA1199, we employed the Wnt/TCF-luciferase reporter assay. As shown in Fig. 5a, hMSC-KIAA1199^down^ exhibited significant decrease in Wnt/TCF activity compared with non-target siRNA controls. hMSC-KIAA1199^over^ exhibited opposite changes with enhanced Wnt/TCF-luciferase reporter activity (Fig. 5b). Consistent with the results, mRNA gene expression levels of Wnt/β-catenin responsive genes, such as DKK2, Axin2, TNFRSF19–2, and NKD1 were all significantly inhibited in KIAA1199^down^ cell while enhanced in KIAA1199^over^ (Fig. 5c). Moreover, adding KIAA1199-CM rescued the inhibition of Wnt/TCF reporter activity in KIAA1199^down^ hMSCs (Fig. 5d). We further examined several signaling pathways known to be important in cell motility^39^. Western-blot analysis showed that phosphorylation of P38 and active β-catenin were decreased in KIAA1199^down^ hMSCs, while were enhanced in KIAA1199^over^ hMSCs (Fig. 6a). Similar changes were observed in the phosphorylation of GSK3β (Thr390) (Fig. 6a). To confirm the association between P38 changes and hMSCs migration, inhibition of p38 signaling by P38 small molecule inhibitor SB203580 reduced the KIAA1199-induced cell migration (Fig. 6b). Furthermore, to corroborate the role of Wnt signaling, we treated the cells with Wnt signaling inhibitor WiKi4 that resulted in reduced the KIAA1199-induced cell migration in a dose dependent fashion (Fig. 6c).

## Discussion

Identifying factors mediating osteoprogenitor cell mobilization to future bone formation sites during fracture healing is relevant for understanding the biology of bone remodeling and bone formation dynamics, as well as for clinical use of the cells for enhancing bone regeneration. In the current study, we identified a secreted factor KIAA1199 which is expressed in the human bone microenvironment by the motile osteoprogenitor cells and it enhances stem and osteoprogenitor cell motility and migration. We also demonstrated that KIAA1199 exerts significant changes in cell morphology, actin cytoskeletal dynamics and mediates its effects through cooperative regulation of the canonical Wnt and P38/MAPK signaling (Fig. 6d).

Bone formation during bone remodeling depends on migration of a cohort of osteoprogenitor cells to future bone formation sites. Recent histomorphometric studies that have employed a number of markers to identify the osteoprogenitor cell populations, demonstrated that osteoprogenitor cells correspond to the cells that are defined as ‘reversal cells’ in classical histomorphometries studies^40^. We observed that KIAA1199 is enriched in ‘reversal cells’ in human bone biopsies that are colonizing future bone formation sites. In addition, KIAA1199 is also expressed in their local reservoir of osteoprogenitor cells (canopy cells, bone marrow envelope cells, and bone lining cells)^41^. This pattern of localization support the hypothesis that mobilizing factors are needed to allow osteoprogenitor cells to reach the critical threshold cell density needed for initiating bone formation on eroded surfaces.

We have employed hMSCs as an in vitro model for osteoprogentior cells and the in vitro counterpart of the ‘reversal cells’. In support of this choice is that the in vivo phenotype of reversal cells is characterized by the expression of a number markers e.g., SMA, Runx2, Collagen type III^40^ that are highly expressed in cultured hMSCs^42^. In addition, cultured hMSCs can form mineralized matrix in vitro and form normal lamellar bone in vivo^25^ corroborating that the in vitro cultured hMSC are enriched in osteoprogenitor cells.

KIAA1199 enhanced cell migration of hMSCs in two independent assays. The in vitro scratch assay that tests the effects on cell migration in presence of intact cell-extracellular matrix (ECM) and cell–cell interactions, thus mimicking the migration of cells in vivo^30^. In addition, Boyden chamber transwell migration assay was employed to test cell migration in suspension. The observed enhanced motility and migration of hMSCs by KIAA1199 corroborates previous findings of reported for its role in cancer cell biology. For example, in gastric cancer cell lines, KIAA1199 supported cancer cell migration and invasion and its expression was upregulated in invasive gastric cancer tissues associated with poor prognosis^43^. Similar results were obtained in breast cancer^33^, where KIAA1199 was associated with enhanced breast cancer cell motility and its expression was upregulated in invasive breast cancer associated with in vivo metastases and poor prognosis^33^.

Migration-stimulating signals induce morphologic and cytoskeletal changes^34^. We observed that the presence of KIAA1199 was associated with fibroblast-like cellular morphology, whereas its absence led to cell rounding and reduced F-actin levels. Similar to our findings, overexpressing KIAA1199 in a human SW480 colon cancer cells was associated with changes in cellular morphology from round cell to a flatter epithelial-like morphology^32^. Also, deficiency of KIAA1199 in human breast cancer cell lines MDA-MB-435 or MDA-MB-231 led to changed cell morphology from fibroblast-like to a polarized epithelial-like morphology. This was accompanied by a reorganization of F-actin from stress fibers to a cortical ring-like structure^33^. We have extended these results by providing a possible molecular explanation for the changes in actin cytoskeleton by demonstrating significant changes in the levels of actin depolymerizing factors, including Cofilin1 (CFL1) and Destrin (DSTN)^28,44^. Interestingly, we have previously reported that these factors to mediate significant changes in actin cytoskeleton during osteoblast differentiation of hMSCs and that the increase in p-CFL1, p-LIMK1, and the decreased DSNT were associated with enhanced osteoblast differentiation^28^, which suggests that the observed changes in cell morphology are needed for the bone forming functions of osteoprogenitor cells.

How does KIAA1199 exert its effects on cell motility? We have tested a number of signaling pathways regulating cell motility and migration. Wnt signaling has been reported to play a role in cell migration^45,46^ and it is an important regulator of bone formation in bone microenvironment^47^. We observed that KIAA1199 enhanced Wnt/β-catenin signaling pathway. Similar to our results, KIAA1199 upregulates Wnt/β-catenin signaling in gastric cancer cells and its deficiency reduces β-catenin expression in NCI-N87 and AGS cancer cells^43^. In addition to Wnt signaling, we observed that high levels of p38 were associated with KIAA1199-mediated increased in hMSCs migration. Interaction between Wnt and p38 has been described as regulator for MSCs biology^48^. Wnts are capable of activating p38 MAPK: positive effects of Wnt4 on osteoblastic cell differentiation of MSCs are mediated through cross-talk between Wnt and p38 MAPK signaling^49^; Wnt3a increases active p38 which in turn enhances osteoblast differentiation in C3H10T1/2 mesenchymal cells^50^. On the other hand, P38 MAPK regulated Wnt/beta-catenin through inactivation of GSK or LRP6^51,52^. However, whether a physical interaction exists between Wnt, p38 signaling, and KIAA1199 remain to be determined.

We observed the expression of KIAA1199 in osteoprogenitor cells colonizing eroded surfaces, reflecting the reversal-resorption phase; corroborate a possible role in osteoprogenitor cell migration in vivo. Two previous findings support this hypothesis. First, KIAA1199 has recently been report to depolymerize hyaluronic acid (HA)^53^ and immunohistochemical studies have demonstrated the presence of HA in the endothelium lining of the sinusoids and endostium^54^ similar to the location where we identified KIAA1199-expressing cells. Second, KIAA1199 degrades HA that is induced by inflammatory mediators present in bone microenvironment and upregulated during bone remodeling^55,56^. Our data suggest a model where osteoprogenitor cells are retained in their niche through cell anchorage to matrix HA, probably through CD44 receptor which is highly expressed in osteoprogenitor cells^57^. During the reversal-resorption phase of bone remodeling, high levels of KIAA1199 likely degrades HA, leading to mobilization of osteoprogentior cells. This model suggest a new role for KIAA1199 as an ECM remodeling factor during the reversal-resorption phase that regulates adhesion and migration properties of osteoprogenitor cells. Our data further support the biological role of KIAA1199 in bone biology, as it interacts with Wnt and P38/MAPK singling, important regulators of bone formation and bone remodeling.

## Electronic supplementary material

Supplementary information
