# Supplementary material for: KIAA1199 is a secreted molecule that enhances osteoblastic stem cell migration and recruitment
Source: Cell Death Dis. 2019 Feb 12;10(2):126. doi: 10.1038/s41419-018-1202-9 (PMC6372631; doi:10.1038/s41419-018-1202-9)
Supplement: Supplementary file 1 — Supplementary information [file 41419_2018_1202_MOESM1_ESM.pdf]

Supplementary information

1. Supplemental figure 1 (Fig. S1)

2. Supplemental figure 2 (Fig. S2)

Fig. S1

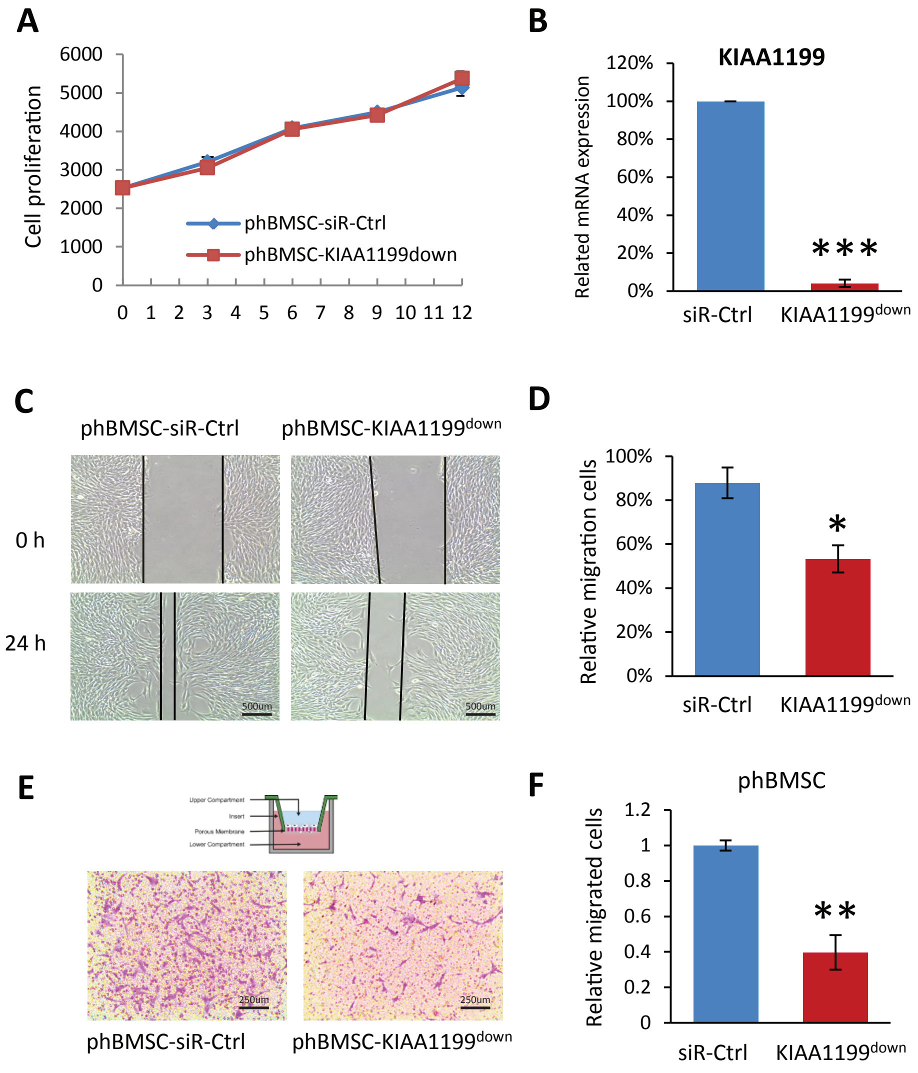

Fig. S1. KIAA1199 regulates primary human bone marrow skeletal (stromal) stem cells (phBMSCs) cell migration. Primary human bone marrow stromal cells were isolated from bone marrow samples from healthy male donors , cells were cultured and passage No. 2 cells were used in the experiment. Cells were seeding and transfected by non-target control siRNA (siR-Ctrl) and siRNA specific for human KIAA1199 (siR-KIAA1199). After 36 hours transfection, cells were subjected to migration tests. (A) Cell poliferation was compared between siR-Ctrl and siR-KIAA1199 phBMSCs. (B) Knockdown efficiency of KIAA1199 in phBMSCs were detected by real time PCR after 36 hours (h) transfection. (C-D) *in vitro* scratch assay . Photomicrographs were obtained at the same position of culture dish after 24 hours (h) incubationin 0.2% fetal bovine serum (FBS) cultured medium (C) and the ability of the cells to "heal the wound" (migrated area) was determined as a ratio of cell covered area per total area by Image-J® program (n ≥ 3) (D). (E-F) Boyden Chamber trans-well cell migration assay were performed and cell migration was measured after 20 hours. The migrated cells were calculated by Image-J program (n ≥ 3). *P<0.05; **P<0.01; ***P<0.001.

Fig. S2

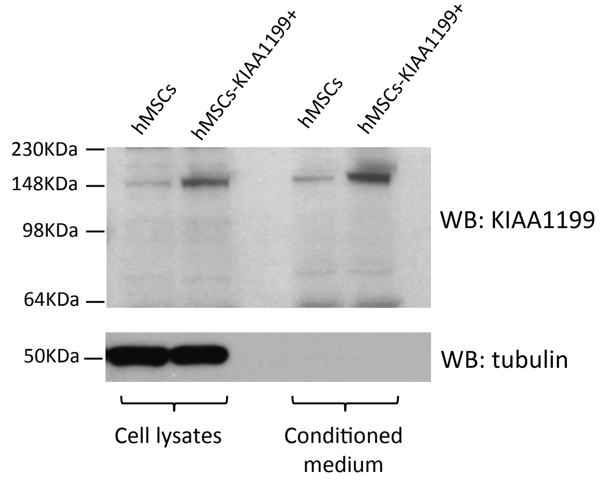

Fig. S2. KIAA1199 is expressed and secreted by human skeletal (stromal) stem cells (hMSCs). Western blot analysis was performed to detect KIAA1199 in cell lysates and conditioned culturing medium from hMSCs and hMSCs overexpression KIAA1199 (hMSCs-KIAA1199+).
